# Intraoperative real-time recognition of dissectible layers by artificial intelligence is useful in laparoscopic inguinal hernia repair

**DOI:** 10.1007/s00464-026-13241-2

**Published:** 2026-08-20

**Authors:** Kazuhito Mita, Shumei Mineta, Kunihiko Takahashi, Mayu Shimaguchi, Taku Shimada, Takashi Sakai, Naoyuki Toyota, Minoru Hatano, Tsuyoshi Toyota, Junichi Sasaki

**Affiliations:** Department of Surgery, Tsudanuma Central General Hospital, 1-9-17 Yatsu, Narashino City, Chiba Japan

**Keywords:** Artificial intelligence, Real-time artificial intelligence navigation, Transabdominal preperitoneal inguinal hernia repair, EUREKAα

## Abstract

**Background:**

Accurate identification of anatomical landmarks such as loose connective tissue, nerves, the vas deferens, and microvessels is essential for transabdominal preperitoneal inguinal hernia repair (TAPP). However, this skill largely depends on the surgeon’s experience. EUREKAα (Anaut Inc., Tokyo, Japan), an artificial intelligence (AI)-based surgical support system, was approved in Japan in April 2024 to provide real-time intraoperative recognition and visualization of loose connective tissue.

**Methods:**

Of the 508 TAPP procedures performed between February 2020 and December 2025, 54 were carried out using real-time AI navigation (RAIN), whereas 454 were performed using conventional techniques. Patient characteristics, surgical outcomes, and postoperative complications were statistically analyzed to evaluate the effectiveness and safety of RAIN. Propensity score matching (PSM) was conducted.

**Results:**

The RAIN group showed significantly shorter operative times (48.6 *vs*. 38.6 min) compared with the conventional group, with no significant differences in postoperative complications. After PSM, 53 matched pairs of patients for each group. Mean operative time (49.2 *vs*. 38.5 min) was significantly shorter in the RAIN group compared to the conventional group.

**Conclusions:**

Real-time intraoperative AI surgical support in TAPP was associated with shorter operative time without increasing postoperative complications. As the clinical use of EUREKAα is still in its early stages, its full value will be more clearly defined through future studies.

**Graphical Abstract:**

**Figure:**
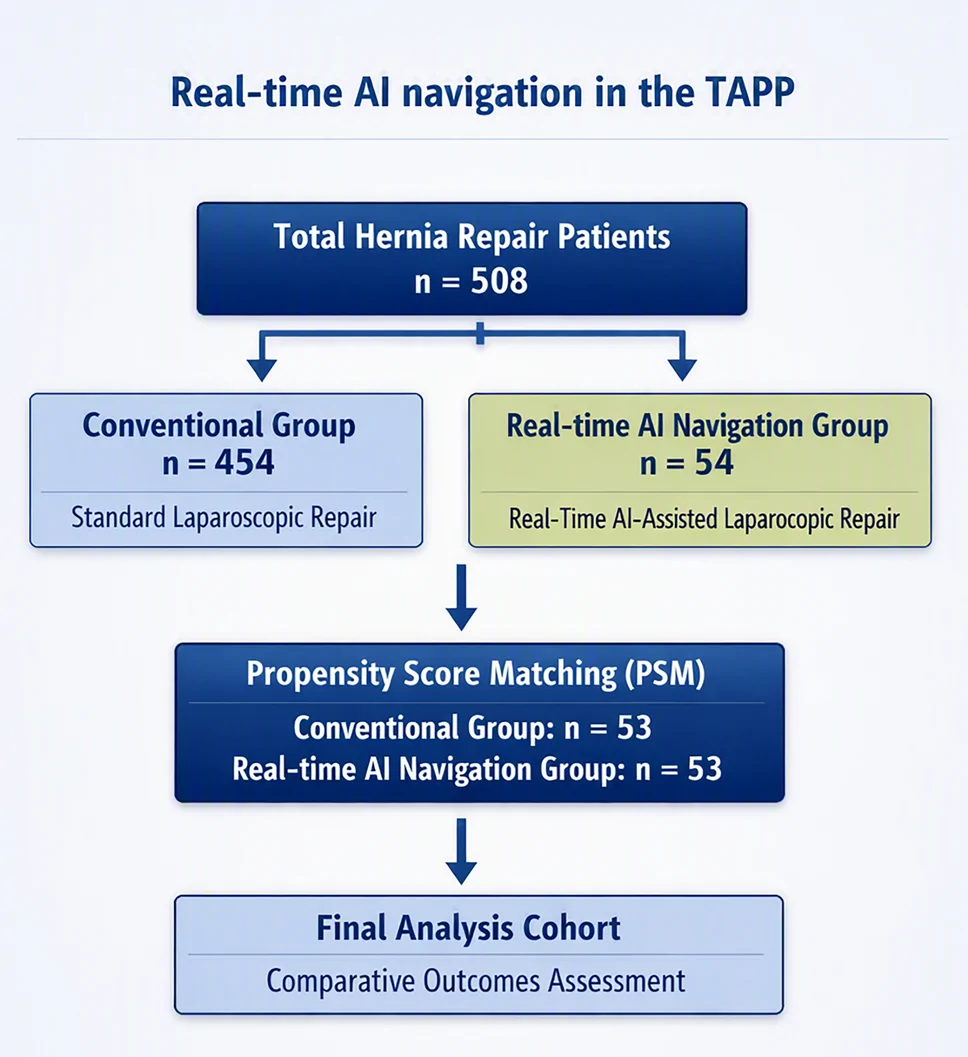

**Supplementary Information:**

The online version contains supplementary material available at https://doi.org/10.1007/s00464-026-13241-2.

Minimally invasive surgery in gastrointestinal surgery has advanced markedly in recent years, driven by improvements in endoscopic equipment and robotic technology [1–3]. In laparoscopic surgery, surgeons must accurately identify fine anatomical landmarks using high-resolution camera images; however, this ability largely depends on the surgeon’s experience [4–6]. For smoother laparoscopic procedures, camera assistants are also required to recognize anatomical landmarks with a high degree of precision.

The artificial intelligence (AI) surgical support program developed by Kumazu et al. can accurately recognize and display loose connective tissue as dissectible layers, as well as other anatomical landmarks such as nerves, the pancreas, ureters, the vas deferens, and microvessels [7–11]. Both qualitative and quantitative evaluations have demonstrated high recognition accuracy for these landmarks. The prototype of this AI program, developed by Anaut Inc., is called EUREKA (Anaut Inc., Tokyo, Japan) and has been used for research and educational purposes; however, its intraoperative real-time use has not been approved. Several reports have described the educational effects and accuracy of EUREKA for medical students and residents [10–12].

EUREKAα (Anaut Inc., Tokyo, Japan), developed based on EUREKA, is an AI surgical support program capable of real-time intraoperative recognition and visualization of loose connective tissue. Qualitative evaluation of EUREKAα for the recognition and visualization of loose connective tissue showed a Dice coefficient of 0.46 and a recall of 0.53, as previously reported by Kumazu et al. [8]. The formulas for calculating the Dice coefficient and recall are as follows (the higher, the better):

$${\text{Dice }} = \frac{TP}{{TP + \frac{1}{2}\left( {FP + FN} \right)}},\,{\mathrm{Recall}} = \,\frac{TP}{{TP + FN}}$$

where TP: true positive, FP: false positive, FN: false negative.

In April 2024, EUREKAα received approval from the Japanese Ministry of Health, Labor and Welfare as the world’s first program-type medical device to provide real-time visual support to surgeons during surgery. With this approval, surgeons and camera assistants can now perform procedures using EUREKAα.

Previous reports on AI surgical support programs have focused mainly on educational effects and recognition accuracy; however, evaluations of real-time intraoperative use in relation to surgical outcomes have not been published.

We introduced EUREKAα into our clinical practice and have performed laparoscopic surgery with real-time recognition and visualization of loose connective tissue.

In transabdominal preperitoneal inguinal hernia repair (TAPP), accurate identification of dissectible loose connective tissue is the most critical skill required by the surgeon. Proper recognition and dissection of loose connective tissue enable appropriate exposure, including the myopectineal orifice, and help prevent intraoperative nerve injury, bleeding, and damage to the vas deferens [13].

The aim of this study was to evaluate the impact of real-time intraoperative recognition and visualization of loose connective tissue using an AI surgical support program on surgical outcomes in TAPP, with particular focus on its effectiveness and safety.

## Materials and methods

### Surgical procedures

**Fig. 1:**
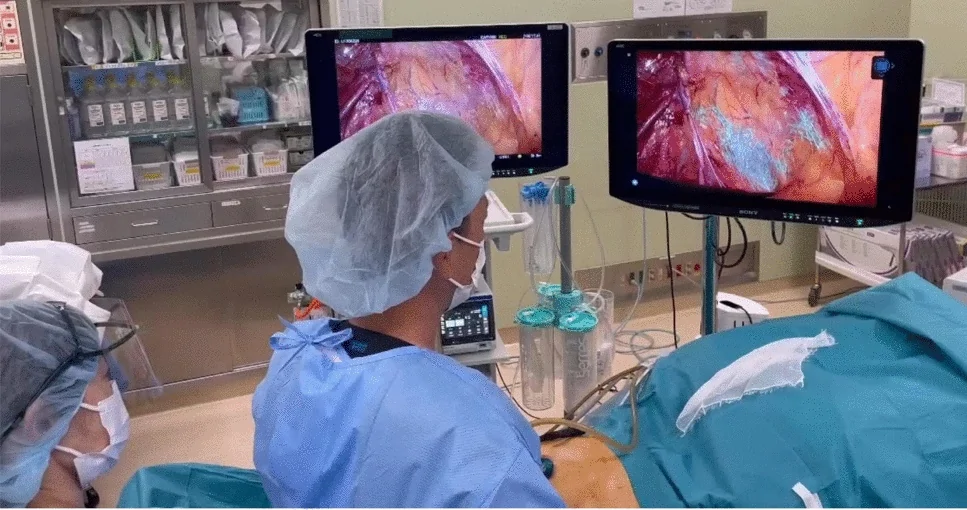
Operating room setup during surgery. The procedure is performed while referencing the output images (recognized images) from EUREKAα displayed on the monitor

In this study, laparoscopic inguinal hernia repair using the TAPP approach was performed with real-time support from the AI surgical support program EUREKAα. EUREKAα was installed on a general-purpose workstation, with endoscopic image input via a serial digital interface cable and output to a medical monitor in the operating room. Figure 1 shows the operating room setup during surgery. The submonitor connected to EUREKAα was positioned as the primary display, and surgery was performed while referencing the recognized output images. The VISERA ELITE2 (Olympus, Tokyo, Japan) endoscopic system was used.

During surgery, the surgeon shared the areas and locations of loose connective tissue highlighted in light blue by EUREKAα with the camera assistant while performing dissection. In some cases, the surgeon verbally explained the dissected area during the procedure.

**Fig. 2:**
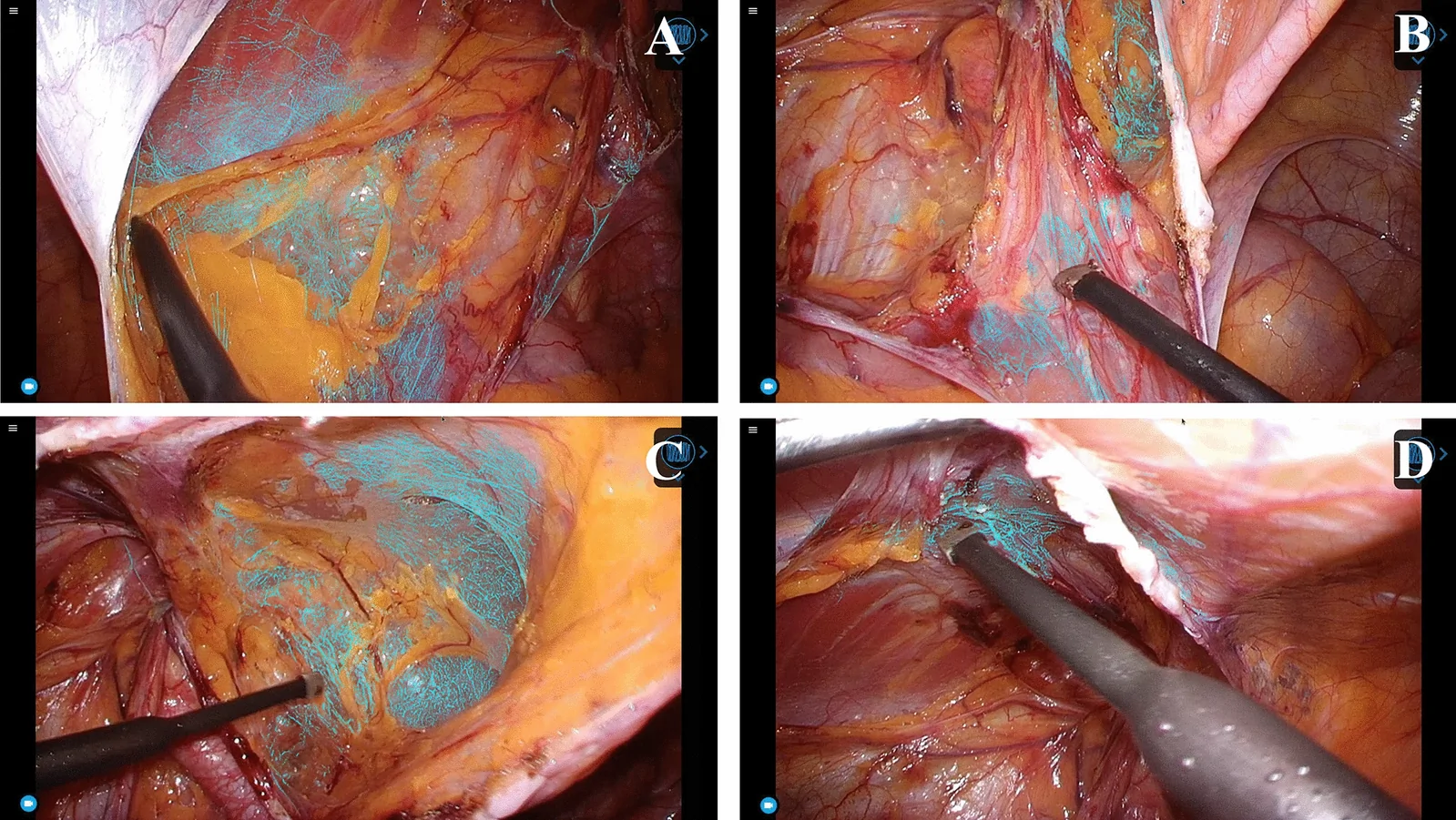
Intraoperative display of loose connective tissue recognized by EUREKAα. Recognized loose connective tissue is highlighted in light blue. **A** lateral aspect, **B** area around the testicular vessels and vas deferens, **C** medial aspect, **D** ventral dissection

All operations were performed by a single surgeon certified by the endoscopic surgical skill qualification system (ESSQS) of the Japan Society for Endoscopic Surgery [14]. The electronic supplementary materials include a video showing EUREKAα recognizing the dissection layer (Fig. 2).

### Patients

This study was approved by the Research Ethics Committee of the Tsudanuma Central General Hospital (Chiba, Japan) (approval number: TCGH-2412) and conducted in accordance with the ethical standards of the Helsinki Declaration. Informed consent was obtained from all patients, and study details were published on the institutional website using an opt-out method. The study included 508 cases of TAPP procedures performed between February 2020 and December 2025, of which 54 were conducted using EUREKAα. Patients who underwent simultaneous bilateral surgery, emergency surgery for an incarcerated hernia, or redo surgery were excluded from this study.

Cases performed without AI surgical support were designated as the conventional TAPP group, whereas those performed with real-time AI support were designated as the TAPP with real-time AI navigation (RAIN) group. The allocation to the RAIN group was not randomized. EUREKAα was introduced in our institution in May 2025, and all consecutive TAPP procedures performed after its implementation were included in the RAIN group. Procedures performed before the implementation constituted the conventional group. Patient characteristics and surgical outcomes were compared between the groups. Postoperative complications were classified as Clavien–Dindo Grade 1 or higher.

### Statistical analysis

**Fig. 3:**
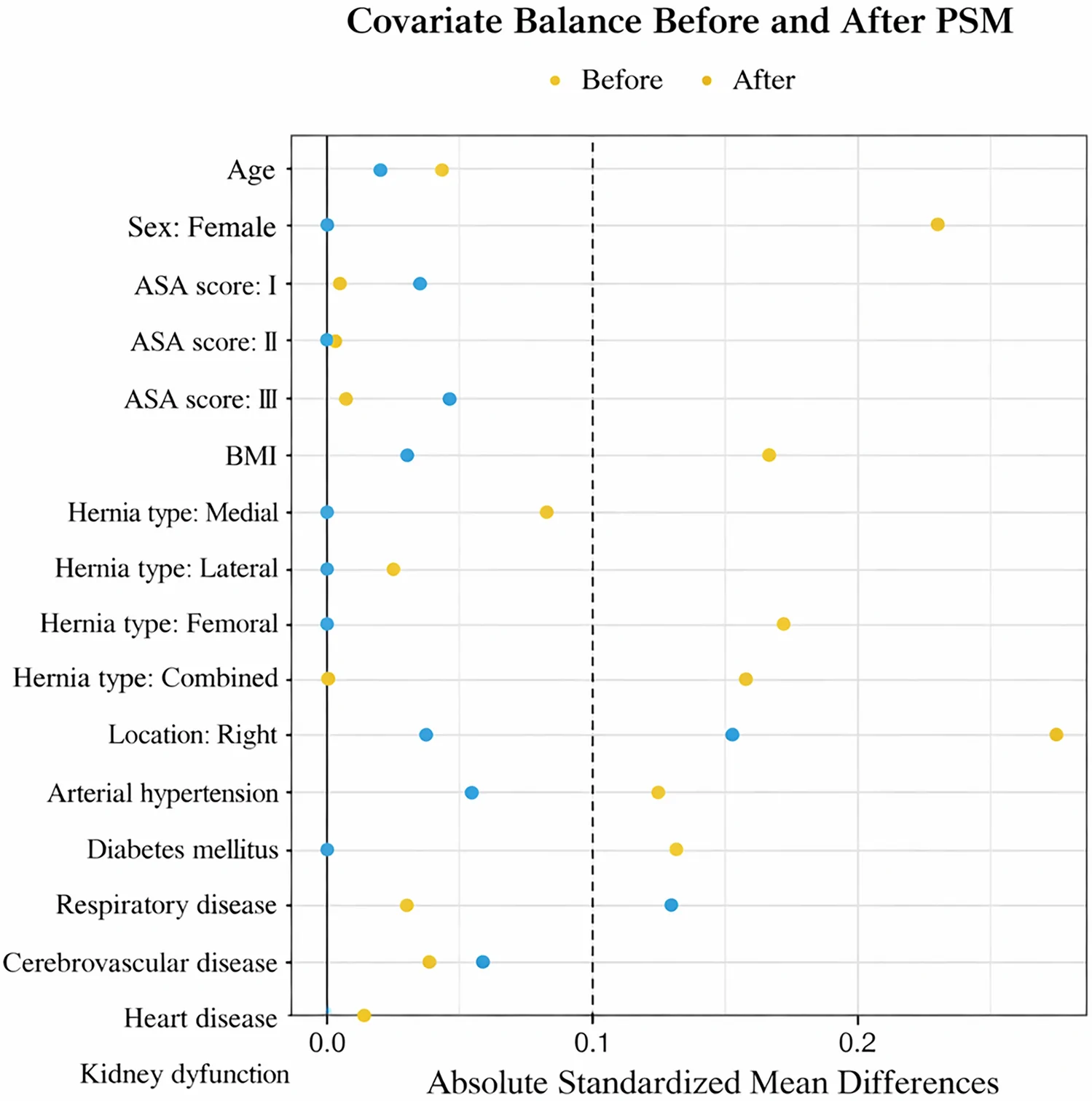
Covariate balance before and after PSM

All statistical analyses were performed using R version 4.4.2 (http://www.R-project.org). Continuous variables were assessed for normality (Shapiro–Wilk test) and homogeneity of variance (Levene’s test) and are expressed as mean ± SD or median (interquartile range), as appropriate. Between-group comparisons before matching were performed using Welch’s t-test or the Wilcoxon rank-sum test for continuous variables and Fisher’s exact test for categorical variables. To adjust for baseline imbalances, propensity score matching (PSM) was performed using 12 covariates (including age, sex, body mass index, American Society of Anesthesiologists score, hernia type, location, and six major comorbidities), with 1:1 nearest neighbor matching and a caliper width of 0.2 of the standard deviation of the logit of the propensity score. Covariate balance was evaluated using standardized mean differences (SMDs), with SMD < 0.2 indicating acceptable balance (Tables. 1 and 2 and Fig. 3). After matching, paired comparisons were performed using the paired t-test or Wilcoxon signed-rank test for continuous variables and McNemar’s exact test for categorical variables; Bowker’s test was used for variables with three or more categories. All tests were two-sided, and *P* < 0.05 was considered statistically significant.

**Table 1:** Patient characteristics before propensity score matching

Variable	Conventional TAPP (*n* = 454), *n* (%)	TAPP with RAIN (*n* = 54), *n* (%)	*P*-value	SMD
Age (years), (mean ± SD)	68.4 ± 14.2	67.8 ± 12.4		0.044
Sex			0.340	0.229
Male	405 (89.2)	51 (94.4)		
Female	49 (10.8)	3 (5.6)		
ASA physical status score			1.000	0.004
I	228 (50.2)	27 (50.0)		0.004
II	135 (29.7)	16 (29.6)		0.002
III	91 (20.0)	11 (20.4)		0.008
BMI	23.1 ± 3.0	23.6 ± 3.3	0.248	0.166
Hernia type			0.601	
Medial	101 (22.2)	14 (25.9)		0.084
Lateral	331 (72.9)	40 (74.1)		0.027
Combined	10 (2.2)	0 (0.0)		0.174
Femoral	12 (2.6)	0 (0.0)		0.159
Location			1.000	0.001
Left	202 (44.5)	24 (44.4)		0.001
Right	252 (55.5)	30 (55.6)		0.001
Comorbidity				
Present	219 (48.2)	27 (50.0)	0.886	–
Arterial hypertension	116 (25.6)	21 (38.9)	0.050	0.274
Diabetes mellitus	40 (8.8)	7 (13.0)	0.320	0.124
Respiratory disease	7 (1.5)	0 (0)	1.000	0.132
Cerebrovascular disease	46 (10.1)	5 (9.3)	1.000	0.030
Heart disease	56 (12.3)	6 (11.1)	1.000	0.039
Kidney dysfunction	18 (4.0)	2 (3.7)	1.000	0.014

*ASA* American Society of Anesthesiologists, *BMI* body mass index, *RAIN* real-time artificial intelligence navigation, *SD* standard deviation, *TAPP* transabdominal preperitoneal (inguinal hernia repair), *SMD* standardized mean differences

**Table 2:** Patient characteristics after propensity score matching

Variable	Conventional TAPP (*n* = 53), *n* (%)	TAPP with RAIN (*n* = 53), *n* (%)	*P*-value	SMD
Age (years), (mean ± SD)	67.4 ± 14.7	67.7 ± 12.5	0.912	0.021
Sex			1.000	0.000
Male	50 (94.3)	50 (94.3)		
Female	3 (5.7)	3 (5.7)		
ASA Physical Status score			0.954	
I	26 (49.1)	27 (50.9)		0.038
II	16 (30.2)	16 (30.2)		0.000
III	11 (20.8)	10 (18.9)		0.047
BMI	23.8 ± 3.1	23.7 ± 3.3	0.843	0.031
Hernia type			N/A	
Medial	14 (26.4)	14 (26.4)		0.000
Lateral	39 (73.6)	39 (73.6)		0.000
Combined	0 (0.0)	0 (0.0)		0.000
Femoral	0 (0.0)	0 (0.0)		0.000
Location			0.481	0.152
Left	20 (37.7)	24 (45.3)		
Right	33 (62.3)	29 (54.7)		
Comorbidity				
Present	27 (50.9)	26 (49.1)	1.000	–
Arterial hypertension	21 (39.6)	20 (37.7)	1.000	0.039
Diabetes mellitus	5 (9.4)	6 (11.3)	1.000	0.056
Respiratory disease	0 (0.0)	0 (0.0)	1.000	0.000
Cerebrovascular disease	7 (13.2)	5 (9.4)	0.687	0.130
Heart disease	6 (11.3)	5 (9.4)	1.000	0.060
Kidney dysfunction	2 (3.8)	2 (3.8)	1.000	0.000

*ASA* American Society of Anesthesiologists, *BMI* body mass index, *RAIN* real-time artificial intelligence navigation, *SD* standard deviation, *TAPP* transabdominal preperitoneal (inguinal hernia repair), *N/A* not applicable, *SMD* standardized mean differences

## Results

### Patient characteristics

A total of 508 patients were enrolled and underwent TAPP. Table 1 summarizes the patient characteristics. Comparison between the conventional TAPP group (454 cases) and the TAPP with RAIN group (54 cases) revealed no significant differences in mean age (68.4 *vs*. 67.8 years), sex distribution, American Society of Anesthesiologists score, or body mass index (23.1 *vs*. 23.6; *p* = 0.248). There were also no significant differences in hernia type or location. No significant differences were observed for hypertension, diabetes mellitus, respiratory disease, cerebrovascular disease, heart disease, or renal dysfunction.

After PSM, 53 matched pairs of patients were included for each group. The patient characteristics of the patients after PSM are shown in Table 2.

### Surgical outcomes

Table 3 summarizes the surgical outcomes. Operative time was significantly shorter in the TAPP with RAIN group (48.6 *vs*. 38.6 min; *p* < 0.0001). No significant difference was observed in postoperative complications. Table 4 shows the postoperative outcomes of the two groups after matching. After PSM, operative time (49.2 *vs*. 38.5 min; *p* < 0.0001) was significantly shorter in the TAPP with RAIN group with no significant differences in postoperative complications.

**Table 3:** Surgical outcomes

Variable	Conventional TAPP (*n* = 454)	TAPP with RAIN (*n* = 54)	*P*-value
Operation time, min (mean ± SD)			
Unilateral	48.6 ± 14.1	38.6 ± 6.3	< 0.0001
Postoperative hospital stay (days), (mean ± SD)	1.1 ± 0.6	1.1 ± 0.5	0.039
Postoperative complications (%)	36 (7.9)	2 (3.7)	0.410
Seroma	24 (5.3)	2 (3.7)	1.000
Bowel obstruction	0 (0.0)	0 (0)	1.000
Surgical site infection	2 (0.4)	0 (0)	1.000
Hematoma	8 (1.8)	0 (0)	1.000
Chronic postoperative inguinal pain	1 (0.2)	0 (0)	1.000

*RAIN* real-time artificial intelligence navigation, *SD* standard deviation, *TAPP* transabdominal preperitoneal (inguinal hernia repair)

**Table 4:** Surgical outcomes after propensity score matching

Variable	Conventional TAPP (*n* = 53)	TAPP with RAIN (*n* = 53)	*P*-value
Operation time, min (mean ± SD)			
Unilateral	49.2 ± 16.4	38.5 ± 6.4	< 0.0001
Postoperative hospital stay (days), (mean ± SD)	1.1 ± 0.4	1.2 ± 0.5	0.753
Postoperative complications (%)	2 (3.8)	2 (3.8)	1.000
Seroma	1 (1.9)	2 (3.8)	1.000
Bowel obstruction	0 (0.0)	0 (0.0)	1.000
Surgical site infection	1 (1.9)	0 (0.0)	1.000
Hematoma	0 (0.0)	0 (0.0)	1.000
Chronic postoperative inguinal pain	0 (0.0)	0 (0.0)	1.000

*RAIN* real-time artificial intelligence navigation, *SD* standard deviation, *TAPP* transabdominal preperitoneal (inguinal hernia repair)

## Discussion

This study demonstrated that real-time intraoperative use of the AI surgical support program in TAPP was associated with shorter operative times, with no increase in postoperative complications after PSM. Several factors likely contributed to these improved outcomes:

### Improvement in surgeons’ recognition of anatomical landmarks

During surgery, surgeons must process extensive information regarding anatomical landmarks, and this ability varies among individuals, typically developing through extensive experience. RAIN supports surgery by recognizing and highlighting specific anatomical landmarks. Our study findings suggest that EUREKAα enhanced surgeons’ recognition of key structures. Although prior studies focused on educational use rather than intraoperative support, several reports have demonstrated the benefits of using the AI surgical support program (EUREKA) for learning anatomical landmark recognition.

Okamura et al. reported that training with EUREKA reduced discrepancies between dissection lines drawn by medical students and those by certified surgeons in robot-assisted gastrectomy [12]. Ryu et al. reported that EUREKA improved nerve recognition by resident surgeons during laparoscopic left colectomy [10]. Kinoshita et al. found that learning with EUREKA significantly improved nerve recognition accuracy in robot-assisted rectal cancer surgery [11].

Highlighting specific structures plays an important role in educational feedback by focusing learners’ attention and clarifying areas for improvement, thereby enhancing effectiveness. Real-time highlighting during surgery provides clear guidance and alerts, helping to reduce misrecognition and errors.

This study demonstrated that real-time intraoperative use of AI can confer educational benefits to surgeons. Similar benefits may be expected for other anatomical landmarks, including nerves and microvessels.

### Enhanced collaboration between surgeons and camera assistants

Morris et al. suggested that AI can support decision-making and intraoperative navigation and that real-time highlighting of anatomical structures may be particularly useful during surgery. They further noted that, through multimodal integration—such as tracking gaze to understand surgeons’ intentions—AI can augment human capabilities [15].

Similarly, Torring et al. proposed that visual cues, such as highlighting, can promote intuitive and efficient collaboration among surgical team members. They emphasized that, especially in complex surgical procedures, synchronizing perceptions and clarifying roles are key factors for success [16].

In this study, RAIN’s highlighting of loose connective tissue facilitated visual information sharing between surgeons and camera assistants, making instructions and goals easier to communicate and improving team collaborative efficiency. Explicit identification of target anatomical landmarks enabled shared and synchronized attention, enhanced situational awareness, and supplemented nonverbal communication.

Improved recognition and collaborative efficiency between surgeons and camera assistants were associated with shorter operative times. Expanding the range of recognized anatomical landmarks may further improve surgical precision; reduce operative time.

As the first report of surgical outcomes using a real-time AI surgical support program, this study has several limitations. First, because the allocation was based on the time of introduction of the AI system, selection bias cannot be fully excluded. The reduction in operative time may partially reflect accumulated experience, improved team coordination, or institutional standardization. This learning-curve effect cannot be fully excluded. Second, the lack of either qualitative or quantitative evaluations comparing the real-time AI surgical support program with conventional surgical methods. In particular, the specific contribution of EUREKAα’s ability to recognize and highlight loose connective tissue to the enhanced precision of surgical maneuvers remains to be assessed. Furthermore, such assessment should be performed by ESSQS-certified surgeons. Furthermore, factors such as the proportion of time the operative field is centered on the monitor, the rate of discrepancies between the surgical field’s movement, and the direction of camera movement must also be assessed. Third, the matched sample size of 53 pairs limits the ability to detect differences in uncommon postoperative complications. Fourth, all procedures were performed by a single ESSQS-certified surgeon. While this minimizes performance variability, it limits generalizability. The benefit of EUREKAα may differ between novice and experienced surgeons. Fifth, because the BMI of our cohort was relatively low, the performance of EUREKAα in high BMI patients, where adipose tissue may obscure anatomical landmarks, remains unclear. Sixth, the present study did not include redo TAPP cases. Given the difficulty of identifying tissue planes in reoperative fields, evaluating the utility of EUREKAα in such settings is an important future direction. Taken together, these limitations highlight the need for further studies in larger and more diverse populations, ideally through multicenter prospective randomized trials, to clarify the effectiveness, safety, and generalizability of real-time AI surgical support.

The present study demonstrates technical feasibility and improved intraoperative visualization with EUREKAα, along with a reduction in operative time. Real-time AI assisted surgery is still at an early stage, and its true value is expected to be demonstrated in future studies.

## Supplementary Information

Below is the link to the electronic supplementary material.

Supplementary file1 (MP4 77136 KB)

Supplementary file2 (MP4 87905 KB)
